# Inter-rater reliability of functional MRI data quality control assessments: A standardised protocol and practical guide using pyfMRIqc

**DOI:** 10.3389/fnins.2023.1070413

**Published:** 2023-02-03

**Authors:** Brendan Williams, Nicholas Hedger, Carolyn B. McNabb, Gabriella M. K. Rossetti, Anastasia Christakou

**Affiliations:** ^1^Centre for Integrative Neuroscience and Neurodynamics, University of Reading, Reading, United Kingdom; ^2^School of Psychology and Clinical Language Sciences, University of Reading, Reading, United Kingdom; ^3^Cardiff University Brain Research Imaging Centre (CUBRIC), School of Psychology, College of Biomedical and Life Sciences, Cardiff University, Cardiff, United Kingdom

**Keywords:** fMRI, resting state fMRI, task fMRI, quality control, inter-rater reliability

## Abstract

Quality control is a critical step in the processing and analysis of functional magnetic resonance imaging data. Its purpose is to remove problematic data that could otherwise lead to downstream errors in the analysis and reporting of results. The manual inspection of data can be a laborious and error-prone process that is susceptible to human error. The development of automated tools aims to mitigate these issues. One such tool is pyfMRIqc, which we previously developed as a user-friendly method for assessing data quality. Yet, these methods still generate output that requires subjective interpretations about whether the quality of a given dataset meets an acceptable standard for further analysis. Here we present a quality control protocol using pyfMRIqc and assess the inter-rater reliability of four independent raters using this protocol for data from the fMRI Open QC project (https://osf.io/qaesm/). Data were classified by raters as either “include,” “uncertain,” or “exclude.” There was moderate to substantial agreement between raters for “include” and “exclude,” but little to no agreement for “uncertain.” In most cases only a single rater used the “uncertain” classification for a given participant’s data, with the remaining raters showing agreement for “include”/“exclude” decisions in all but one case. We suggest several approaches to increase rater agreement and reduce disagreement for “uncertain” cases, aiding classification consistency.

## Introduction

Functional magnetic resonance imaging (fMRI) data are inherently multi-dimensional with many potential sources of artefacts that can lead to spurious results (Power et al., 2012; Van Dijk et al., 2012). Therefore, ensuring data are of sufficient quality for analysis is an essential step in the processing of fMRI data. This is especially important for large multi-site studies such as the Adolescent Brain Cognitive Development study (Casey et al., 2018), and the Human Connectome Project (Van Essen et al., 2013), where time required to perform detailed, manual screening of individual data can quickly become intractable. To address this, many quality control tools and pipelines now exist to help users make informed decisions about quality in their datasets (Marcus et al., 2013; Esteban et al., 2017; Alfaro-Almagro et al., 2018). These tools–which automate part of the quality control process–aim to decrease the time taken to assess data quality, minimise the amount of prior knowledge needed to make informed decisions, and reduce errors during assessment.

Several tools currently exist for assessing the quality of fMRI data, including MRIQC (Esteban et al., 2017), and Visual QC (Raamana, 2018). This list also includes pyfMRIqc, which we developed at the Centre for Integrative Neuroscience and Neurodynamics (CINN), University of Reading (Williams and Lindner, 2020). Many of our neuroimaging facility users at CINN are Ph.D. students and early career researchers, who join a community that prioritises practical training and learning opportunities. As part of this commitment, we develop software which is user-friendly and empowers individuals to become confident and informed researchers. pyfMRIqc helps users to make informed decisions about the quality of their data by generating various image quality metrics and presenting them in an easily interpretable way in a visual report. pyfMRIqc also has extensive online documentation that describes to users how these plots are generated and what they show, and aids their interpretation with examples. Users of pyfMRIqc can generate these reports with minimal programming experience, requiring only a single line of code to run the software and without the need for using containerised environments for generating output. As part of the work presented here, we additionally developed a piece of software, “cinnqc,” which we used to automate the minimal pre-processing and curation of data for pyfMRIqc, and to identify cases where data deviate from the expected acquisition parameters for the dataset.

Previous reports describe the use of inter-rater reliability for the quality assessment of structural imaging data (Backhausen et al., 2016; Esteban et al., 2017; Rosen et al., 2018; Benhajali et al., 2020). For instance, Benhajali et al. (2020) developed a method for quickly assessing the registration of T1 weighted images to standard MNI space. Raters included citizen scientists who had no previous experience with MRI data, as well as expert raters. Their protocol resulted in good reliability, particularly with respect to which images were deemed to fail quality assessment, between expert raters, with citizen scientists also showing agreement. The study therefore demonstrated that this straightforward approach for assessing registration quality was consistent between individuals with different skill levels. Another protocol assessed for reliability between raters was presented by Backhausen et al. (2016), who aimed to provide a workflow for the quality control assessment of T1 images both during and after image acquisition to maximise useful sample size. Images were classified into three categories (pass, check, fail), and these three categories were associated with significant differences in cerebral cortex, left amygdala, and total grey matter volume estimations. Reliability between two raters for the three classification categories was high [intra-class correlation coefficient (α = 0.931)], in line with results from Rosen et al. (2018), who found good consistency between expert raters when a three category rating system was used (although notably concordance was significantly lower when using five categories). Lastly, Esteban et al. (2017) demonstrated fair to moderate agreement between two raters when assessing the quality of T1 data from the ABIDE dataset. These studies demonstrate that reasonable reliability can be expected of subjective decisions about the quality of structural imaging data, particularly when three categories are used to classify data. However, in the case of functional data, and despite its potential utility, inter-rater reliability has not been similarly evaluated to help understand the consistency of subjective decisions about data quality. To assess whether experienced raters are reliable in their classifications of functional data quality across datasets, we used data from the fMRI Open QC project,^1^ which included data with different acquisition parameters from multiple sites.

We assess the inter-rater reliability of fMRI data quality assessments for task-based and resting state data. We describe quantitative and qualitative criteria for classifying data quality, present a quality control protocol for assessing raw fMRI data quality using pyfMRIqc, assess reliability between four independent raters using this protocol, and provide example cases of different data quality issues using output from pyfMRIqc. Raters classified data into one of three assessment categories, “include,” “uncertain,” or “exclude.” Using our protocol, we find moderate to substantial reliability between raters, particularly for “include” and “exclude” decisions, but less agreement between raters for the uncertain classification.

## Materials and methods

### Participants

#### Imaging data participants

Imaging data from 129 subjects were included. Each subject had a T1 weighted high-resolution anatomical image, and a single-band echo-planar imaging (EPI) image for either task-based or resting state functional magnetic resonance imaging (fMRI) acquisition. Task-based fMRI data were included for 30 subjects. Resting-state fMRI data were included for 99 subjects; resting-state data originated from five sites, with approximately 20 subjects per site. Data originated from the following publicly available datasets: ABIDE, ABIDE-II, Functional Connectome Project, and OpenNeuro (Biswal et al., 2010; Di Martino et al., 2014; Markiewicz et al., 2021). Data from each site were treated as separate datasets for the purpose of performing quality assessment. The expected acquisition parameters for data from each site are summarised in Table 1. The data presented here are available on the Open Science Framework page of the fMRI Open QC project (see text footnote 1).

**TABLE 1:** Expected acquisition parameters for subjects in each site in the main dataset.

Subjects	Modality	Voxel size (mm)	Matrix	Volumes	TR (s)
sub-001 → sub-030	T1w	1 × 1 × 1	176 × 256 × 256	1	
	EPI	3 × 3 × 4	64 × 64 × 34	242	2
sub-101 → sub-120	T1w	1 × 1 × 1	256 × 200 × 256	1	
	EPI	2.67 × 2.67 × 3	96 × 96 × 47	156	2.5
sub-201 → sub-220	T1w	1 × 1 × 1	160 × 256 × 256	1	
	EPI	3 × 3 × 3.840789	80 × 80 × 38	150	2
sub-301 → sub-316	T1w	0.976562 × 1.2 × 0.976562	256 × 182 × 256	1	
	EPI	1.5625 × 1.5625 × 3.1	128 × 128 × 45	162	2.5
sub-401 → sub-423	T1w	1 × 1 × 1	256 × 200 × 256	1	
	EPI	2.667 × 2.667 × 3	96 × 96 × 47	123	2.5
sub-701 → sub-720	T1w	1 × 1 × 1	192 × 256 × 256	1	
	EPI	3 × 3 × 3.51	64 × 64 × 39	198	2.5

T1w modality is the high-resolution T1 weighted anatomical image. EPI modality is the functional (BOLD) task-based (sub-001 → sub-030) and resting state (sub-101 → sub-720) echo-planar images. TR is the time taken in seconds to acquire a single volume of EPI data.

#### Quality control raters

Quality control assessments were completed by four independent raters (BW, NH, CBM GMKR), who were all postdoctoral research fellows, and all raters had previous experience in quality assessment, processing and analysis of functional neuroimaging data. Two raters (BW and GR) had previously used pyfMRIqc to perform quality assessment of fMRI data. Additionally, BW was involved in the development of pyfMRIqc. Each rater reviewed data for 104 of the 129 subjects, using outputs from cinnqc and pyfMRIqc. Subject assignment ensured at least four subjects from each site were reviewed by all four raters, and every other subject was reviewed by three raters. Assignments were also balanced so that the proportion of overlapping cases was equal across raters (see Supplementary Data Sheet 1 for details of rater assignments).

### Data processing

Minimal pre-processing of anatomical T1 weighted and functional EPI data was performed using the FSL toolbox (version 6.0) from the Oxford Centre for Functional MRI of the Brain (FMRIB’s Software Library^2^) (Jenkinson et al., 2012). Data pre-processing, curation, and quality control was automated using “cinnqc.”^3^ cinnqc provides wrapper scripts for executing and curating output from FSL pre-processing functions (e.g., motion correction, registration, and brain extraction), and also generating pyfMRIqc reports for minimally pre-processed data. To pre-process data, the T1 image was skull stripped using the Brain Extraction Tool (Smith, 2002), then grey matter, white matter, and cerebrospinal fluid tissue segmentation was performed using FMRIB’s Automated Segmentation Tool (Zhang et al., 2001). Functional EPI data were motion corrected with MCFLIRT (Jenkinson et al., 2002), using affine transformations to align the first volume of functional data with each subsequent volume. Functional EPI and anatomical T1 data were then co-registered using the epi_reg function,^4^ and a linear affine transformation was used to convert a brain extracted mask of the T1 anatomical image to functional EPI space using FMRIB’s Linear Image Registration Tool (Jenkinson and Smith, 2001; Jenkinson et al., 2002). The brain mask in functional EPI space was then re-binarised using a threshold of 0.5. Image quality metrics and plots were generated using pyfMRIqc (Williams and Lindner, 2020) to aid data quality assessment, e.g., the identification of artefacts that were participant-, sequence-, technique-, or tissue-specific. pyfMRIqc was run with the following input arguments: -n < motion corrected EPI data >, -s 25, -k < brain extracted mask in functional space > -m < motion parameter output from MCFLIRT >.

### Resources

Ubuntu 20.04.4 LTS

FSL version 6.0 (see text footnote 2).

Anaconda 4.10.1

Python 3.8.8

• cinnqc 0.1.0
• easygui 0.98.3
• matplotlib 3.3.4
• nibabel 3.2.1
• numpy 1.20.1
• pandas 1.2.4

### Quality assessment protocol

Raters were given the following instructions before beginning quality assessment:

The following criteria need to be used to classify all images^5^ :

• Include–no quality assessment issues that indicate the dataset is problematic.
• Uncertain–some quality assessment issues that makes the inclusion of dataset marginal.
• Exclude–quality assessment issues that mean the data should not be included.

Each image classified as either “uncertain” or “exclude” should include an explanation of why the given classification was made. Please be as descriptive as possible when explaining your decision-making.

Quality assessment decision-making should be supported by the output produced by cinnqc and pyfMRIqc. cinnqc and pyfMRIqc derivatives can be found online in the directories /cinnqc/ examples/{fmriqc-open-qc-task, fmriqc-open-qc-rest-100, fmriqc-open-qc-rest-200, fmriqc-open-qc-rest-300, fmriqc-open-qc-rest-400, fmriqc-open-qc-rest-500, fmriqc-open-qc-rest-600, fmriqc-open-qc-rest-700}/derivatives/cinnqc/of the cinnqc GitHub page (see text footnote 3).

#### Quantitative data assessment

Quantitative quality assessment criteria for T1 and EPI data based on acquisition parameters and derived metrics from the data are summarised in Table 1. Thresholds for absolute and relative motion, as calculated using MCFLIRT, are given to limit its effect on data quality. Motion thresholds are defined in Table 2 and are summarised in the pyfMRIqc report. Yet, even motion that is sub-threshold could still impact data quality. Qualitative data assessment should be carried out to check whether any motion incidents coincide with a problematic change in signal. Temporal signal to noise (TSNR, referred to as SNR in pyfMRIqc) is calculated as mean intensity divided by the standard deviation of voxels (25th centile mean intensity) outside the brain-extracted mask in functional space. It is calculated by pyfMRIqc on minimally pre-processed data. Slice-wise TSNR should be checked in the pyfMRIqc report, and potentially problematic slices should be followed up using qualitative assessments. Field of view, number of volumes, and scans are checked using cinnqc, and a file with the suffix *_notes.txt is generated to describe any potential issues. Note, some voxel dimensions may appear to be different due to rounding, but if they are equal to 2 decimal places then subjects do not need to be excluded. T1 and EPI data should have whole brain coverage, which includes the cerebral cortex and subcortical brain regions (but not necessarily the cerebellum). A summary of quantitative assessment criteria can be found in Table 2, and a summary of the expected acquisition parameters can be found in Table 1.

**TABLE 2:** Quantitative criteria for determining dataset inclusion/exclusion.

Criteria	Exclusion criteria
Motion	Any relative movements > Voxel size More than 5 relative movements > 0.5 mm^1^ Max absolute motion > 2 mm (1.5 mm is marginal)^1^
Slice-wise SNR	< 99 (99 → 150 is marginal)^1^*
Consistent voxel sizes	No^2^ (to 2d.p.)
Consistent number of volumes	No^2^
Consistent number of scans in the dataset	No^3^
T1w whole brain coverage	No^4^
EPI whole brain coverage in the mean image of the pyfMRIqc report and the first volume	No^4^

*Some slices will return slice-wise TSNR values of NaN. NaN values are returned because the slice does not have any voxels that SNR are calculated for; if this is the case, then the presence of these NaN values should not be used for the purpose of exclusion. Some slices will include a large proportion of non-brain voxels which will have lower values relative to brain voxels decreasing the slice-wise TSNR mean. If this is the case then use your discretion in your assessment of slice-wise TSNR.

^1^: Center for Brain Science, Harvard University (https://cbs.fas.harvard.edu/facilities/neuroimaging/investigators/mr-data-quality-control/);

^2^: Human Connectome Project (Marcus et al., 2013);

^3^: BIDS standard (Gorgolewski et al., 2016);

^4^: UK Biobank (Alfaro-Almagro et al., 2018).

#### Qualitative data assessment

pyfMRIqc generates a number of plots and tables that can be helpful in the qualitative assessment of data. Mean and slice-wise scaled squared difference (SSD) is calculated by squaring the difference in voxel intensity between consecutive volumes, and dividing by the global mean squared difference. In the QC plots section, mean and slice-wise SSD graphs can be used to identify global, and slice-wise changes in signal intensity, respectively. SSD is also plotted alongside the global normalised mean voxel intensity, normalised SSD variance, plus absolute and relative motion to visualise relationships between changes in SSD, signal intensity, and motion. Further, mean, minimum, and maximum SSD is plotted slice-wise to determine whether issues are present in specific slices.

The plot of the “Mean voxel time course of bins with equal number of voxels” is generated by binning voxels into 50 groups, based on their mean intensity, and calculating the mean intensity for voxels in each bin for each volume. Bins are ordered top-down from lowest mean intensity voxels (non-brain/cerebrospinal fluid) to highest (grey matter, then white matter voxels). This plot enables easy visualisation of signal variance and was originally described by Power (2017), where further information can also be found.

The “Masks” plot can be helpful in indicating whether there were issues during acquisition or processing (such as brain extraction and/or registration of T1 and EPI data). For instance, there may be many brain voxels that are not highlighted in blue. If this is the case, then scans should be carefully checked for signal distortion (described below), or processing steps may need to be manually re-run with adjusted input parameters. Poor registration (for instance, misalignment of gross anatomical structures including brain surface, or grey matter/white matter/cerebrospinal fluid boundaries) may be indicative of other data quality issues.

The “Variance of voxel intensity” plot visualises the variance in signal in each voxel over the timeseries of the functional run. The png image given in the pyfMRIqc report is thresholded (voxel intensities are divided into 1,000 equal width bins, and the intensity of the highest bin with at least 400 voxels is used) to aid visualisation, however a nifti version of the image is also included which is unthresholded. This nifti image is useful for more in-depth investigation if there are potential quality issues or the figure appears problematic. The “Sum of squared scaled difference over time” plot presents the voxel-wise sum of SSD over the functional run. Similarly to the “Variance of voxels intensity” plot, we applied a threshold for the png figure for readability (sum of squared scaled differences are divided into 50 equal width bins, and the upper threshold of the fifth bin is used), but the nifti image does not have a threshold.

To inspect data for signal distortion, load T1 images from the subject’s BIDS directory; for EPI images, load the image with the suffix *_example-func.nii.gz from the subject’s cinnqc BIDS derivative directory, and the mean voxel intensity nifti file from pyfMRIqc. If visual abnormalities are present, this could impact the signal (e.g., image distortion, signal loss, artefacts such as ringing or ghosting), or processing (e.g., brain extraction, registration, motion correction) of T1 or EPI data. To determine if this is the case, the plots from pyfMRIqc can be used to aid subject classification. Detailed explanations for interpreting pyfMRIqc plots and tables can be found in the pyfMRIqc User Manual.^6^ A summary of qualitive assessment criteria can be found in Table 3.

**TABLE 3:** Qualitative criteria for determining dataset inclusion.

Criteria	Threshold
Aberrant pyfMRIqc output	Plots or tables that indicate problematic EPI data, supported by visual inspection of functional data
T1w signal distortion	Visual abnormalities in the acquisition of the T1w image, such as ringing artefacts that would impair registration to standard template
EPI signal distortion	Visual abnormalities in the mean image of the pyfMRIqc report or the first volume of the fMRI data that would impair registration to standard template
Atypical brain structure	Morphology that would impair registration to standard template (pathological or non-pathological).

### Rater calibration and reliability assessment

Each rater independently assessed and classified subjects using the quality assessment protocol described above. To ensure quality assessment criteria were interpreted consistently, BW used the quality assessment protocol to identify exemplar subjects for issues and presented these training cases to the other raters (Table 4).

**TABLE 4:** Example cases that were used during the calibration session for raters before independently assessing the whole dataset.

Subject	Include	Uncertain	Exclude	Notes
sub-013			1	Many volumes with relative movement > 0.1. Motion events around volumes 65 and 205 appear to cause global decrease in signal
sub-103	1			Peak in SSD between volumes 95–100 looks like its driven by eye movement
sub-207			1	More than 5 relative motion events > 0.5. Max absolute movement is marginal

Fleiss’ kappa (Fleiss, 1971) was calculated using the “irr” package in R (Gamer et al., 2019) to assess pair-wise and category-wise inter-rater reliability between raters; to correct for multiple comparisons we used the Holm method to control the family-wise error rate using the “p.adjust” function in R (Holm, 1979; R Core Team, 2020). We chose to use Fleiss’ kappa instead of Cohen’s kappa, because Fleiss’ kappa also allows us to determine how similar pairs of raters are across classifications by calculating category-wise agreement. We used the criteria described by Landis and Koch (1977) to interpret Fleiss’ kappa using the following benchmarks to describe the strength of agreement: poor agreement < 0.00; slight agreement 0.00–0.20; fair agreement 0.21–0.40; moderate agreement 0.41–0.60; substantial agreement 0.61–0.8; almost perfect agreement 0.81–1.00. Overall agreement across raters and categories was calculated using Krippendorff’s alpha (Krippendorff, 1970), which is useful as a measure of overall agreement because it is not restricted by the number of raters or the presence of missing data in the sample (Hayes and Krippendorff, 2007). Krippendorff’s alpha and bootstrap 95% confidence intervals (1,000 iterations, sampling subjects with replacement) were calculated in R using scripts from Zapf et al. (2016).

## Results

Each subject was categorised as either “include” (rater one: 68, rater two: 73, rater three: 80, rater four: 74), “uncertain” (rater one: 10, rater two: 12, rater three: 3, rater four: 9), or “exclude” (rater one: 26, rater two: 19, rater three: 21, rater four: 20) by the four raters. Overall percentage agreement between raters is summarised in Table 5.

**TABLE 5:** Overall percentage agreement between raters for “include”/“uncertain”/“exclude” assignments.

	Rater 1	Rater 2	Rater 3	Rater 4
Rater 1	–	78.481	75.949	75.641
Rater 2	78.481	–	75.949	83.333
Rater 3	75.949	75.949	–	84.615
Rater 4	75.641	83.333	84.615	–

**FIGURE 1:**
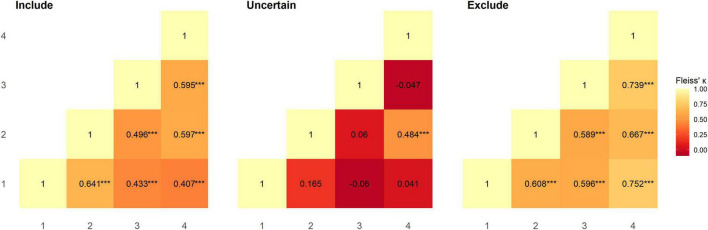
Reliability between pairs of raters for each category was assessed using Fleiss’ Kappa. Agreement between raters at significantly greater than chance level is denoted as ***p < 0.001.

Inter-rater reliability between pairs of raters was calculated using Fleiss’ Kappa; overall agreement between all pairs of raters was moderate and significantly greater than chance level (rater 1–2: κ = 0.536, *z* = 6.143, *p* < 0.001; rater 1–3: κ = 0.437, *z* = 4.639, *p* < 0.001; rater 1–4: κ = 0.456, *z* = 5.17, *p* < 0.001; rater 2–3: κ = 0.448, *z* = 5.071, *p* < 0.001; rater 2–4: κ = 0.596, *z* = 6.818, *p* < 0.001; rater 3–4: κ = 0.578, *z* = 6.022, *p* < 0.001). Category-wise Kappa for all raters was moderate and substantial for “include” and “exclude” assignments respectively and was significantly greater than chance level (“include”: κ = 0.514, *z* = 6.661, *p* < 0.001; “exclude”: κ = 0.731, *z* = 9.472, *p* < 0.001). However, this was not the case for “uncertain” assignments, where agreement between raters was slight (κ = 0.013, *z* = 0.166, *p* = 1.0). We also calculated Fleiss’ Kappa category-wise for pairs of raters (Figure 1). All raters had moderate to substantial agreement, and performed at significantly greater than chance level for “include” (rater 1–2: *z* = 5.697 *p* < 0.001; rater 1–3: *z* = 3.849 *p* < 0.001; rater 1–4: *z* = 3.591 *p* < 0.001; rater 2–3: *z* = 4.409 *p* < 0.001; rater 2–4: *z* = 5.269 *p* < 0.001; rater 3–4: *z* = 5.253 *p* < 0.001) and “exclude” (rater 1–2: *z* = 5.405 *p* < 0.001; rater 1–3: *z* = 5.3 *p* < 0.001; rater 1–4: *z* = 6.645 *p* < 0.001; rater 2–3: *z* = 5.231 *p* < 0.001; rater 2–4: *z* = 5.887 *p* < 0.001; rater 3–4: *z* = 6.525 *p* < 0.001) assignments, but not for “uncertain” (rater 1–2: *z* = 1.471 *p* = 1.0; rater 1–3: *z* = −0.537 *p* = 1.0; rater 1–4: *z* = 1.0 *p* = 0.716; rater 2–3: *z* = 0.529 *p* = 1.0; rater 2–4: *z* = 4.272 *p* < 0.001; rater 3–4: *z* = −0.415 *p* = 1.0) assignments (Figure 1). Overall agreement in the dataset, as assessed using Krippendorff’s alpha was 0.508 [95% bootstrap confidence intervals (0.381, 0.615)]; removing instances where “uncertain” was assigned increased Krippendorff’s alpha to 0.694 [95% bootstrap confidence intervals (0.559, 0.802)]. In total, at least two raters categorised 97 subjects as include, 6 subjects as uncertain, and 26 subjects as exclude. Supplementary Table 1 summarises the subject-wise group majority classification (“include”/“uncertain”/“exclude”).

### QC “exclude” criteria examples

#### Data acquisition artefacts

**FIGURE 2:**
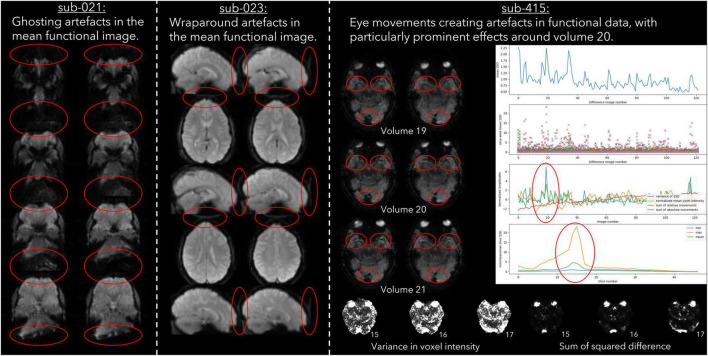
Example cases of three different types of data acquisition artefacts detected using output from pyfMRIqc.

Imaging acquisition artefacts were identified in five subjects by at least one rater. These issues included ghosting (aliasing), ringing, and wraparound artefacts (Table 6). In Figure 2 we present these three artefacts, with the relevant output from pyfMRIqc used to identify the issue. For the first subject, ghosting (aliasing) in the mean functional image from pyfMRIqc was detected (Heiland, 2008). This can be detected visually as the presence of spurious signal outside the perimeter of the head. In the second case, wraparound of the functional signal was detected in the mean functional image (Arena et al., 1995). Wraparound can be detected when part of the head is partially occluded by the field of view. In this case, the most posterior portion of the head appears instead in the anterior portion of the image and is most noticeable visually on axial and sagittal slices. The third case contained in-plane artefacts in the data due to eye movements (McNabb et al., 2020). In this case, both the variance in voxel intensity, plus a peak in the maximum and sum of the scaled squared difference in affected slices (particularly slices 15–17) indicated the presence of physiologically unrelated changes in signal. These effects are especially pronounced around volumes 19–21, where there is a peak in the variance of the sum of squared difference. A video of flickering in affected slices is included in Supplementary Video 1.

**TABLE 6:** Number of datasets excluded by single, majority, and all raters for each of the relevant exclusion reasons.

	Single rater	Majority raters	All raters
Abnormal brain morphology	2	0	0
Aliasing	1	2	0
Global signal	10	1	0
Incorrect acquisition parameters	4	0	0
Motion	9	4	17
Non-whole brain coverage	1	0	0
Ringing artefact	1	0	0
SNR	5	1	0
Unidentified artefact	4	0	0
Wraparound artefact	1	0	0

In all cases where raters excluded a subject, the rater also provided notes explaining their reasons for exclusion. Here, these notes are categorised into groups, and the number of times a single, majority (two of three when three raters were assigned or two/three when four raters were assigned) or all raters mentioned that category in their notes is reported. Note that raters could give multiple reasons for excluding a subject, which means that agreement for exclusion could be based on different reasons. Subject and category-wise frequencies for single, majority, and all raters, as well as the number of raters excluding each subject are included in Supplementary Data Sheet 2.

#### Motion

**FIGURE 3:**
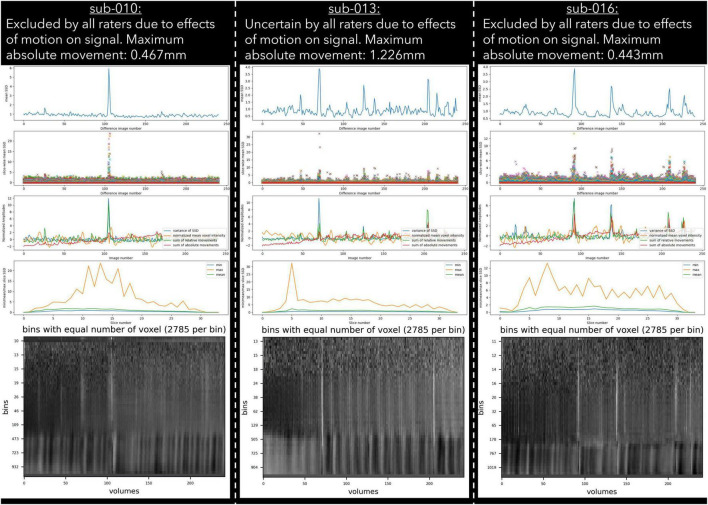
Example cases of three different types of motion artefacts detected using output from pyfMRIqc. One subject (sub-013) was classified as uncertain by all raters, while sub-010 and sub-016 were classified as exclude by all raters.

**FIGURE 4:**
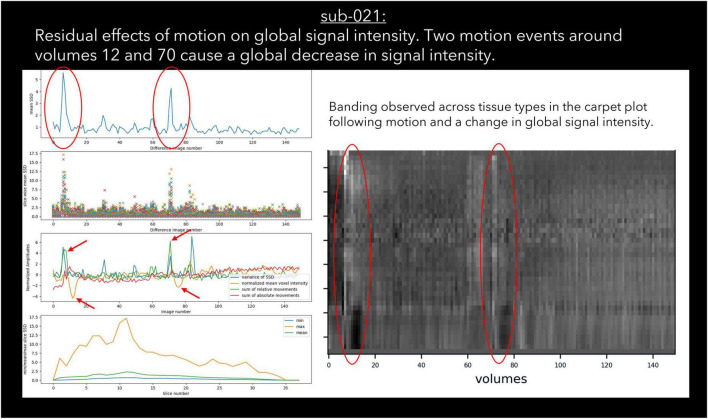
An example case of global signal loss following motion detected using output from pyfMRIqc.

30 datasets were classified as “exclude” by at least one rater with issues relating to motion described in the notes (Table 6). Of these cases, 17 exceeded acceptable values set out in our quantitative criteria for absolute and relative motion (Table 2). The remaining cases were classified as “exclude” based on the residual effects of motion upon the data, despite the quantitative measure of motion being sub-threshold (Friston et al., 1996). This includes decreases in global signal coinciding with the onset of motion events (Figure 4), plus peaks in scaled squared difference and banding in the binned carpet plot (Figure 3; Power, 2017).

#### Signal loss

Sudden changes in global signal can be assessed in several ways using pyfMRIqc. For instance, Figure 4 demonstrates when motion artefacts lead to a sudden decrease in global signal (Power et al., 2017). The onset of head motion around volumes 12 and 70, identified by the peaks in the mean and variance of the scaled squared difference plus the sum of relative and absolute movements, is immediately followed by a decrease in the normalised mean voxel intensity of around two standard deviations for approximately ten volumes (Figure 4). Banding is also present in the binned carpet plot, where sudden changes in signal coincide with changes in intensity across all bins. Six of the reviewed subjects were reported as having SNR related issues by at least one of the four raters, and eleven were reported as having global signal issues (Table 6).

#### Atypical brain structure

**FIGURE 5:**
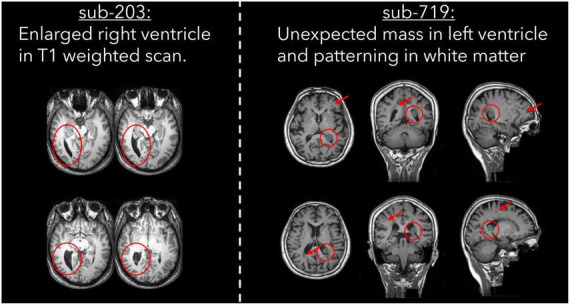
T1 weighted images for two cases of atypical brain structure that were present in the dataset.

Two subjects were excluded by one rater due to the presence of atypical brain structure in the T1 weighted anatomical image (Table 6). Both cases are detailed in Figure 5, with one subject having a right ventricle that was enlarged and covering greater than both the extent of the left ventricle and where we would typically expect the ventricle to cover. The second subject had an unexpected mass in their left ventricle, and hypointensities in white matter across the whole brain. We are unable to comment on the clinical relevance of these anatomical features as none of the authors have clinical expertise.

### Uncertain cases

27 subjects were classified as “uncertain” by at least one rater; “uncertain” was used as a classification by a single rater for 21 subjects, and more than one rater for six subjects. For the subjects classified as “uncertain” by one rater, the other two/three raters gave the same classification (“include”/“exclude”) for 20 of the 21 subjects; one subject received one “include,” one “uncertain,” and one “exclude” classification. For the remaining six cases where more than one rater classified subjects as “uncertain,” the notes for four subjects indicated the presence of issues related to residual motion that were below our threshold, while the notes for the other two subjects indicated the presence of possible pathology in the T1 image and aliasing in functional data. Lastly, only one dataset was rated as “uncertain” by all raters (Figure 4), with raters “uncertain” about the effects of sub-threshold residual motion on the data.

## Discussion

This work aimed to describe a protocol for assessing the quality of raw task-based and resting state fMRI data using pyfMRIqc, and to assess the reliability of independent raters using this protocol to classify data with respect to whether it meets an acceptable standard for further analysis. We used data from the fMRI Open QC Project [(see text footnote 1), data were derived from ABIDE, ABIDE-II, Functional Connectome Project, and OpenNeuro (Biswal et al., 2010; Di Martino et al., 2014; Markiewicz et al., 2021)]. Overall, we found moderate agreement between raters, and moderate to substantial category-wise agreement between raters for include/exclude classifications. Poor to moderate category-wise agreement was found for the uncertain classification, with reliability at significantly greater than chance level for only one pair of raters. Krippendorff’s alpha for the include/exclude categories across all raters was sufficient to tentatively accept the raters’ classifications were reliable (Krippendorff, 2004, p. 241). We also provide examples for different types of quality issues that were identified in the dataset.

For the “uncertain” classification we found that there was a lack of reliability between raters, with two pairs of raters having negative κ values, indicating no agreement (McHugh, 2012), and a further three pairs having coefficients close to 0. The lack of reliability between raters for the “uncertain” classification appears to be driven by the uncertainty of a single rater for a given subject. Of the 27 subjects rated “uncertain” by any rater, 21 (78%) were not rated “uncertain” by the other raters. Of the 6 subjects rated “uncertain” by more than one rater, uncertainty related to concerns about motion (*N* = 4), aliasing (*N* = 2), and possible pathology (*N* = 2). This included one subject (sub-013) who was classified by all raters as “uncertain” due to residual effects of motion, yet other similar subjects (e.g., sub-010 and sub-016) were unanimously classified as “exclude” despite having visually similar plots, and less maximum absolute motion (Figure 3). In our quantitative exclusion criteria (Table 2), we give explicit thresholds for absolute and relative movement events, and though 17/30 excluded data sets were excluded by at least one rater due to exceeding our quantitative movement thresholds, 13/30 were excluded based on qualitative assessment of movement effects on data quality. These thresholds are relatively arbitrary, and despite being a helpful heuristic, they did not appear to capture all cases where motion had an adverse effect on data. pyfMRIqc counts the number of relative motion events > voxel size, 0.5 mm and 0.1 mm, and though we set our thresholds for the number of relative motion events > voxel size and 0.5 mm based on previous guidelines,^7^ we did not set a threshold for motion events > 0.1, but < 0.5 mm. In cases where motion was sub-threshold but still an issue, persistent but small motion events could negatively impact data as we did not include a threshold for small (0.1 < motion < 0.5) motion events. Nevertheless, it is worth mentioning that data reviewed here by raters was only minimally pre-processed and that approaches such as ICA-based denoising (Pruim et al., 2015), the inclusion of motion parameters in a model (Friston et al., 1996), and removing volumes affected by motion (Power et al., 2012) can, and often are used during data pre-processing to decrease the negative effects of motion on the signal in fMRI data. However, though these approaches are helpful for cleaning data that may otherwise be discarded, we feel that consensus guidelines for (un)acceptable levels of motion are needed to improve consistency within the neuroimaging community, in the same way the BIDS standard (Gorgolewski et al., 2016) has been widely adopted as the *de facto* data formatting structure.

It is important to note that despite the data only being minimally pre-processed, the purpose of pyfMRIqc is not to determine whether data processing steps worked as expected, but to assess the quality of the data itself. We motion corrected data so that our metrics (e.g., scaled squared difference) are calculated for contiguous voxels in time and space but we do not directly measure whether all physical motion was corrected for. Brain extraction, spatial normalisation, distortion correction, and denoising, are all commonly used and important pre-processing steps in the pipeline of fMRI data analysis, and the efficacy of these pre-processing steps should also be checked as part of a robust analysis pipeline for ensuring data quality. Therefore, the output generated by pyfMRIqc should be treated as one part of a broader data processing procedure. Additionally, because the image quality metrics generated by pyfMRIqc have no absolute reference – that is they cannot be compared to a reference value since there is no ground truth – the detection of data quality issues is dependent on individual interpretation. One way to address this issue is by generating a database of reference values to aid outlier detection. This is the process used by MRIQC, which crowdsources image quality metrics to generate population-level distributions (Esteban et al., 2017). However, we are currently unable to generate these distributions with pyfMRIqc.

Cognitive biases may also influence subjective decision-making about the quality of fMRI data. The acquisition and preparation of an fMRI dataset involves great economic and time cost, and researchers may be motived more by these sunk costs to minimise loss from their own data than from secondary datasets. People tend to be loss averse (Kahneman and Tversky, 1979), and the thought of “wasting” the resources put into acquiring the dataset could bias individuals to perceive data quality issues as less problematic than if the data were collected independently. For instance, Polman (2012) found that people are less loss averse when making decisions for others compared to themselves, and that this reduction of loss aversion may be due to a decreasing effect of cognitive bias on decision making. Compared with others, people also disproportionately value things they have created themselves (Norton et al., 2012), and may therefore be reluctant to discard data they perceive as having value. Reappraisal is one strategy that can be used to decrease loss aversion (Sokol-Hessner et al., 2009, 2013), and could improve decision-making by changing the perspective of discarding data from a waste of spent resource to a way of maximising ability to detect effects and improve data quality. The adoption of open research practices, such as the preregistration of data quality control procedures and acceptable thresholds could also decrease the risk of biases influencing decision-making, while at the same time reducing questionable research practices more generally (Niso et al., 2022). However this has not yet been widely adopted in the neuroimaging community (Borghi and Gulick, 2018).

There are several limitations in the protocol and software as presented here. Firstly, our finding that often only a single rater classified a dataset as “uncertain” suggests that the quality control protocol presented (which is published unedited from its pre-assessment state), lacked nuance for interpreting edge cases that would otherwise have been classified as either “include” or “exclude.” Given that pyfMRIqc was initially designed to aid decision-making about the quality of raw/minimally pre-processed fMRI data, we suggest that future users err on the side of caution with respect to marking datasets for exclusion, and first fully pre-process data using their pipeline of choice and then determine whether this had a positive impact and reduced data quality issues. Second, cinnqc, and by extension pyfMRIqc, do not formally quantify the success of the minimal pre-processing steps. When designing software for users with minimal programming experience, prioritising ease of use over functionality can reduce the freedom of more advanced users. For instance, brain extraction currently uses default arguments in FSL to identify brain and non-brain tissue (Smith, 2002). This process can sometimes exclude brain voxels (particularly at the boundary of the brain), or include non-brain voxels in the brain extracted image. However, these issues can be ameliorated *via* optional arguments that change the default values, but this requires fine tuning on a per-subject basis, or the use of other software like HD-BET or ANTsX (Isensee et al., 2019; Tustison et al., 2021). A method for integrating these features would improve the computational reproducibility of the quality control procedure, as currently users would need to generate these files separately and use the cinnqc nomenclature to integrate output with the rest of the pipeline. A third limitation is that pyfMRIqc does not currently provide visualisation for distributions of “no-reference” image quality metrics. As previously mentioned, MRIQC currently crowdsources these values from users by default to generate robust distributions (Esteban et al., 2017). Though pyfMRIqc does not currently have the userbase to make this an effective method for identifying outliers at the population level, visualising the distribution of these values for at least the group level would help users to make more informed decisions about the quality of data they have in their sample. Future versions of pyfMRIqc would be improved by focusing on including these features in the software, and could potentially integrate reference values from the MRIQC Web-API for equivalent metrics in a similar way to how MRIQCEPTION^8^ works.

In summary, we present a quality control protocol for pyfMRIqc (Williams and Lindner, 2020), implement it on data from the fMRI Open QC project (see text footnote 1), and assess its reliability using four independent raters. Data were classified by each rater as either “include,” “uncertain,” or “exclude,” based on the protocol and output generated by pyfMRIqc and cinnqc, which automated minimal pre-processing, data curation, and identification of deviated acquisition parameters in the dataset. Our results indicate that our reliability between raters was good for “include” and “exclude” decisions, with κ values that ranged from moderate to substantial agreement. However, coefficients for the “uncertain” classification demonstrated little reliability between raters, and below chance level for all but one pair of raters. Furthermore, we found that in all but one cases where only one rater used the “uncertain” classification the other raters agreed with each other. We suggest that improvements in agreement between raters could be made by consulting sample-wide distributions of image quality metrics, increasing the clarity of the quality control protocol, and implementing further separate pre-processing steps before reassessing the data and deciding whether or not to exclude them.

## Data availability statement

Each rater’s quality control report can be found in Supplementary material as Data Sheets 3–6, and at the University of Reading Research Data Archive https://doi.org/10.17864/1947.000424. pyfMRIqc and cinnqc output can be found on the cinnqc GitHub page https://github.com/bwilliams96/cinnqc.

## Ethics statement

Ethical review and approval was not required for the study on human participants in accordance with the local legislation and institutional requirements. Written informed consent for participation was not required for this study in accordance with the national legislation and the institutional requirements.

## Author contributions

BW: conceptualization, methodology, software, formal analysis, investigation, data curation, writing—original draft, review and editing, visualization, and project administration. NH, CM, and GR: methodology, analysis, and review and editing. AC: resources, supervision, and review and editing. All authors contributed to the article and approved the submitted version.

## Footnotes

1
https://osf.io/qaesm/

2
www.fmrib.ox.ac.uk/fsl

3
https://github.com/bwilliams96/cinnqc

4
https://fsl.fmrib.ox.ac.uk/fsl/fslwiki/FLIRT/UserGuide#epi_reg

5 Additional information about classifications not given in the protocol but which was agreed by raters:

• Include cases would pass all quantitative and qualitative quality control criteria and pyfMRIqc plots or manual inspection of data would not indicate any issues with data.
• Uncertain cases would pass all quantitative quality control criteria, but pyfMRIqc plots or manual inspection of data may indicate marginal issues in the data that could warrant exclusion.
• Exclude cases would fail at least one quantitative quality control criteria, and/or pyfMRIqc plots or manual inspection of data indicate data quality issues that would warrant exclusion.

6
https://drmichaellindner.github.io/pyfMRIqc/

7
https://cbs.fas.harvard.edu/facilities/neuroimaging/investigators/mr-data-quality-control/

8
https://github.com/elizabethbeard/mriqception

## References

[B1] Alfaro-Almagro F. Jenkinson M. Bangerter N. K. Andersson J. L. R. Griffanti L. Douaud G. (2018). Image processing and quality control for the first 10,000 brain imaging datasets from UK Biobank. *Neuroimage* 166 400–424. 10.1016/j.neuroimage.2017.10.034 29079522 PMC5770339

[B2] Arena L. Morehouse H. T. Safir J. (1995). MR imaging artifacts that simulate disease: How to recognize and eliminate them. *Radiographics* 15 1373–1394. 10.1148/radiographics.15.6.8577963 8577963

[B3] Backhausen L. L. Herting M. M. Buse J. Roessner V. Smolka M. N. Vetter N. C. (2016). Quality control of structural MRI images applied using freeSurfer–A hands-on workflow to rate motion artifacts. *Front. Neurosci.* 10:558. 10.3389/fnins.2016.00558 27999528 PMC5138230

[B4] Benhajali Y. Badhwar A. Spiers H. Urchs S. Armoza J. Ong T. (2020). A standardized protocol for efficient and reliable quality control of brain registration in functional MRI studies. *Front. Neuroinformatics* 14:7. 10.3389/fninf.2020.00007 32180712 PMC7059806

[B5] Biswal B. B. Mennes M. Zuo X.-N. Gohel S. Kelly C. Smith S. M. (2010). Toward discovery science of human brain function. *Proc. Natl. Acad. Sci. U.S.A.* 107 4734–4739. 10.1073/pnas.0911855107 20176931 PMC2842060

[B6] Borghi J. A. Gulick A. E. V. (2018). Data management and sharing in neuroimaging: Practices and perceptions of MRI researchers. *PLoS One* 13:e0200562. 10.1371/journal.pone.0200562 30011302 PMC6047789

[B7] Casey B. J. Cannonier T. Conley M. I. Cohen A. O. Barch D. M. Heitzeg M. M. (2018). The adolescent brain cognitive development (ABCD) study: Imaging acquisition across 21 sites. *Dev. Cogn. Neurosci.* 32 43–54. 10.1016/j.dcn.2018.03.001 29567376 PMC5999559

[B8] Di Martino A. Yan C.-G. Li Q. Denio E. Castellanos F. X. Alaerts K. (2014). The autism brain imaging data exchange: Towards a large-scale evaluation of the intrinsic brain architecture in autism. *Mol. Psychiatry* 19:6. 10.1038/mp.2013.78 23774715 PMC4162310

[B9] Esteban O. Birman D. Schaer M. Koyejo O. O. Poldrack R. A. Gorgolewski K. J. (2017). MRIQC: Advancing the automatic prediction of image quality in MRI from unseen sites. *PLoS One* 12:e0184661. 10.1371/journal.pone.0184661 28945803 PMC5612458

[B10] Fleiss J. L. (1971). Measuring nominal scale agreement among many raters. *Psychol. Bull.* 76:378. 10.1037/h0031619

[B11] Friston K. J. Williams S. Howard R. Frackowiak R. S. J. Turner R. (1996). Movement-Related effects in fMRI time-series. *Magn. Reson. Med.* 35 346–355. 10.1002/mrm.1910350312 8699946

[B12] Gamer M. Lemon J. Singh I. F. P. (2019). *irr: Various coefficients of interrater reliability and agreement.* https://CRAN.R-project.org/package=irr (accessed October 14, 2022).

[B13] Gorgolewski K. J. Auer T. Calhoun V. D. Craddock R. C. Das S. Duff E. P. (2016). The brain imaging data structure, a format for organizing and describing outputs of neuroimaging experiments. *Sci. Data* 3:1. 10.1038/sdata.2016.44 27326542 PMC4978148

[B14] Hayes A. F. Krippendorff K. (2007). Answering the call for a standard reliability measure for coding data. *Commun. Methods Meas.* 1 77–89. 10.1080/19312450709336664

[B15] Heiland S. (2008). From A as in Aliasing to Z as in Zipper: Artifacts in MRI. *Clin. Neuroradiol.* 18 25–36. 10.1007/s00062-008-8003-y

[B16] Holm S. (1979). A simple sequentially rejective multiple test procedure. *Scand. J. Stat.* 6 65–70.

[B17] Isensee F. Schell M. Pflueger I. Brugnara G. Bonekamp D. Neuberger U. (2019). Automated brain extraction of multisequence MRI using artificial neural networks. *Hum. Brain Mapp.* 40 4952–4964. 10.1002/hbm.24750 31403237 PMC6865732

[B18] Jenkinson M. Smith S. (2001). A global optimisation method for robust affine registration of brain images. *Med. Image Anal.* 5 143–156. 10.1016/S1361-8415(01)00036-6 11516708

[B19] Jenkinson M. Bannister P. Brady M. Smith S. (2002). Improved optimization for the robust and accurate linear registration and motion correction of brain images. *Neuroimage* 17 825–841. 10.1016/S1053-8119(02)91132-8 12377157

[B20] Jenkinson M. Beckmann C. F. Behrens T. E. Woolrich M. W. Smith S. M. (2012). FSL. *Neuroimage* 62 782–790. 10.1016/j.neuroimage.2011.09.015 21979382

[B21] Kahneman D. Tversky A. (1979). Prospect theory: An analysis of decision under risk. *Econometrica* 47:263. 10.2307/1914185

[B22] Krippendorff K. (1970). Estimating the reliability, systematic error and random error of interval data. *Educ. Psychol. Meas.* 30 61–70. 10.1177/001316447003000105

[B23] Krippendorff K. (2004). *Content analysis: An introduction to its methodology*, 2nd Edn. Thousand Oaks, CA: Sage.

[B24] Landis J. R. Koch G. G. (1977). The measurement of observer agreement for categorical data. *Biometrics* 33 159–174. 10.2307/2529310 843571

[B25] Marcus D. S. Harms M. P. Snyder A. Z. Jenkinson M. Wilson J. A. Glasser M. F. (2013). Human connectome project informatics: Quality control, database services, and data visualization. *Neuroimage* 80 202–219. 10.1016/j.neuroimage.2013.05.077 23707591 PMC3845379

[B26] Markiewicz C. J. Gorgolewski K. J. Feingold F. Blair R. Halchenko Y. O. Miller E. (2021). The openNeuro resource for sharing of neuroscience data. *Elife* 10:e71774. 10.7554/eLife.71774 34658334 PMC8550750

[B27] McHugh M. L. (2012). Interrater reliability: The kappa statistic. *Biochem. Med.* 22 276–282. 10.11613/BM.2012.031 PMC3900052 23092060

[B28] McNabb C. B. Lindner M. Shen S. Burgess L. G. Murayama K. Johnstone T. (2020). Inter-slice leakage and intra-slice aliasing in simultaneous multi-slice echo-planar images. *Brain Struct. Funct.* 225 1153–1158. 10.1007/s00429-020-02053-2 32140847 PMC7166208

[B29] Niso G. Botvinik-Nezer R. Appelhoff S. Vega A. D. L. Esteban O. Etzel J. A. (2022). Open and reproducible neuroimaging: From study inception to publication. *Neuroimage* 263:119623. 10.1016/j.neuroimage.2022.119623 36100172 PMC10008521

[B30] Norton M. I. Mochon D. Ariely D. (2012). The IKEA effect: When labor leads to love. *J. Consum. Psychol.* 22 453–460. 10.1016/j.jcps.2011.08.002

[B31] Polman E. (2012). Self–other decision making and loss aversion. *Organ. Behav. Hum. Decis. Process.* 119 141–150. 10.1016/j.obhdp.2012.06.005

[B32] Power J. D. (2017). A simple but useful way to assess fMRI scan qualities. *Neuroimage* 154 150–158. 10.1016/j.neuroimage.2016.08.009 27510328 PMC5296400

[B33] Power J. D. Barnes K. A. Snyder A. Z. Schlaggar B. L. Petersen S. E. (2012). Spurious but systematic correlations in functional connectivity MRI networks arise from subject motion. *Neuroimage* 59 2142–2154. 10.1016/j.neuroimage.2011.10.018 22019881 PMC3254728

[B34] Power J. D. Plitt M. Laumann T. O. Martin A. (2017). Sources and implications of whole-brain fMRI signals in humans. *Neuroimage* 146 609–625. 10.1016/j.neuroimage.2016.09.038 27751941 PMC5321814

[B35] Pruim R. H. R. Mennes M. van Rooij D. Llera A. Buitelaar J. K. Beckmann C. F. (2015). ICA-AROMA: A robust ICA-based strategy for removing motion artifacts from fMRI data. *Neuroimage* 112 267–277. 10.1016/j.neuroimage.2015.02.064 25770991

[B36] R Core Team. (2020). *R: A language and environment for statistical computing.* Vienna: R Foundation for Statistical Computing.

[B37] Raamana P. R. (2018). *VisualQC: Assistive tools for easy and rigorous quality control of neuroimaging data.* Genèv: Zenodo, 10.5281/zenodo.1211365

[B38] Rosen A. F. G. Roalf D. R. Ruparel K. Blake J. Seelaus K. Villa L. P. (2018). Quantitative assessment of structural image quality. *Neuroimage* 169 407–418. 10.1016/j.neuroimage.2017.12.059 29278774 PMC5856621

[B39] Smith S. M. S. M. S. M. (2002). Fast robust automated brain extraction. *Hum. Brain Mapp.* 17 143–155. 10.1002/hbm.10062 12391568 PMC6871816

[B40] Sokol-Hessner P. Camerer C. F. Phelps E. A. (2013). Emotion regulation reduces loss aversion and decreases amygdala responses to losses. *Soc. Cogn. Affect. Neurosci.* 8 341–350. 10.1093/scan/nss002 22275168 PMC3594725

[B41] Sokol-Hessner P. Hsu M. Curley N. G. Delgado M. R. Camerer C. F. Phelps E. A. (2009). Thinking like a trader selectively reduces individuals’ loss aversion. *Proc. Natl. Acad. Sci. U.S.A.* 106 5035–5040. 10.1073/pnas.0806761106 19289824 PMC2656558

[B42] Tustison N. J. Cook P. A. Holbrook A. J. Johnson H. J. Muschelli J. Devenyi G. A. (2021). The ANTsX ecosystem for quantitative biological and medical imaging. *Sci. Rep.* 11:1. 10.1038/s41598-021-87564-6 33907199 PMC8079440

[B43] Van Dijk K. R. A. Sabuncu M. R. Buckner R. L. (2012). The influence of head motion on intrinsic functional connectivity MRI. *Neuroimage* 59 431–438. 10.1016/j.neuroimage.2011.07.044 21810475 PMC3683830

[B44] Van Essen D. C. Smith S. M. Barch D. M. Behrens T. E. J. Yacoub E. Ugurbil K. (2013). The WU-minn human connectome project: An overview. *Neuroimage* 80 62–79. 10.1016/j.neuroimage.2013.05.041 23684880 PMC3724347

[B45] Williams B. Lindner M. (2020). pyfMRIqc: A software package for raw fMRI data quality assurance. *J. Open Res. Softw.* 8:1. 10.5334/jors.280

[B46] Zapf A. Castell S. Morawietz L. Karch A. (2016). Measuring inter-rater reliability for nominal data – which coefficients and confidence intervals are appropriate? *BMC Med. Res. Methodol.* 16:93. 10.1186/s12874-016-0200-9 27495131 PMC4974794

[B47] Zhang Y. Brady M. Smith S. M. (2001). Segmentation of brain MR images through a hidden Markov random field model and the expectation-maximization algorithm. *IEEE Trans. Med. Imaging* 20 45–57. 11293691 10.1109/42.906424

